# Heat Shock Protein 70 and 90 Genes in the Harmful Dinoflagellate *Cochlodinium polykrikoides*: Genomic Structures and Transcriptional Responses to Environmental Stresses

**DOI:** 10.1155/2015/484626

**Published:** 2015-04-22

**Authors:** Ruoyu Guo, Seok Hyun Youn, Jang-Seu Ki

**Affiliations:** ^1^Department of Life Science, Sangmyung University, Seoul 110-743, Republic of Korea; ^2^Fishery and Ocean Information Division, National Fisheries Research & Development Institute, Busan 619-705, Republic of Korea

## Abstract

The marine dinoflagellate *Cochlodinium polykrikoides* is responsible for harmful algal blooms in aquatic environments and has spread into the world's oceans. As a microeukaryote, it seems to have distinct genomic characteristics, like gene structure and regulation. In the present study, we characterized heat shock protein (HSP) 70/90 of *C. polykrikoides* and evaluated their transcriptional responses to environmental stresses. Both HSPs contained the conserved motif patterns, showing the highest homology with those of other dinoflagellates. Genomic analysis showed that the *CpHSP70* had no intron but was encoded by tandem arrangement manner with separation of intergenic spacers. However, *CpHSP90* had one intron in the coding genomic regions, and no intergenic region was found. Phylogenetic analyses of separate HSPs showed that CpHSP70 was closely related with the dinoflagellate *Crypthecodinium cohnii* and CpHSP90 with other Gymnodiniales in dinoflagellates. Gene expression analyses showed that both *HSP* genes were upregulated by the treatments of separate algicides CuSO_4_ and NaOCl; however, they displayed downregulation pattern with PCB treatment. The transcription of *CpHSP90* and *CpHSP70* showed similar expression patterns under the same toxicant treatment, suggesting that both genes might have cooperative functions for the toxicant induced gene regulation in the dinoflagellate.

## 1. Introduction

Dinoflagellate algae are a eukaryotic protist and are the most important primary producer in aquatic environments. Some species (e.g.,* Alexandrium tamarense*,* Amphidinium carterae*,* Akashiwo sanguinea*,* Cochlodinium polykrikoides*,* Gymnodinium*, and* Karlodinium micrum*) are responsible for harmful algal blooms (HABs), affecting fisheries and creating associated economic loss in aquaculture industries [1]. The ichthyotoxic* Cochlodinium polykrikoides* is one of the most common dinoflagellates that cause HABs, and it has expanded oceanic regions worldwide [2, 3]. Thus, its bloom can cause severe environmental impacts and huge economic losses, due to lots of fish mortalities in aquaculture [4–6]. In the last three decades, the causative organism has been extensively studied in terms of environmental survey, bloom-forming mechanisms, and/or mitigation measures [7–11]; nevertheless, some issues, like toxic mechanisms and cellular gene response, still remained unclear. Molecular study is very useful and crucial for understanding regulation mechanism and molecular characteristics of the causative organism. However, molecular studies of* C. polykrikoides* especially in terms of toxicogenomics and gene regulation are limited so far.

Heat shock proteins (HSPs) are remarkably evolutionary conserved molecular chaperones and are present in all the prokaryotic and eukaryotic organisms. They are distributed into small HSP, HSP60, HSP70, HSP90, and HSP100, depending on their molecular weight and sequence similarity [12]. HSPs have multiple roles, including membrane translocation, protein degradation, protein folding, and repair misfolded proteins, in regulation of protein homeostasis in normal and stressed cells for regulating protein homeostasis in normal and stressed cells [12, 13]. Hence, HSPs are one of the major genes that can be induced and respond to various stressors, and, of them, HSP70 may be firstly induced under stress conditions rather than other HSPs [12]. Moreover, HSP70 and HSP90 are the most conserved and abundant HSPs and are widely involved in the environmental stressors, such as thermal shock, heavy metal, oxidative damage, hypoxia, and xenobiotic chemicals [12, 14–16]. For this reason, either HSP90 or HSP70 is considered as biomarker for environmental monitoring [17, 18]. Furthermore, HSP90 and HSP70 may interact with each other, as well as cooperate with other HSPs or chaperone to regulate multisignal transduction pathway [19–21].

In cases of the dinoflagellates, several of* HSP90* and* HSP70* genes have been identified, but majority of researches have focused on phylogenetic relationships or spliced leader sequence analyses in dinoflagellates [22–25]. Only few studies have investigated the responses of* HSP90* and/or* HSP70* under environmental stress conditions [26–28]. For example, we reported* HSP70/90* from the dinoflagellates* Prorocentrum minimum*, suggesting that both genes play diverse roles in physiological responses of the dinoflagellate [27, 28]. So, it is necessary to discover more molecular information for understanding gene regulation mechanisms in adaptive, survival strategies of dinoflagellates, as well as gene and genomic structures. In the present study, we determined full length sequences of* HSP90* and* HSP70* of the dinoflagellate* C. polykrikoides* and characterized their gene and genomic features. These included analysis of genomic DNA, deduced protein sequences, phylogenetic relationships, and their gene regulation under metal and nonmetal stress conditions as well.

## 2. Materials and Methods

### 2.1. Cell Culture

*C. polykrikoides* was obtained from the National Fisheries Research and Development Institute (NFRDI), Korea. The* C. polykrikoides* cells were cultured in f/2 medium at 20°C in 12:12 h light-dark cycle, with a photon flux density of about 65 *μ*mol photons m^−2^ s^−1^.

### 2.2. RNA Extraction, cDNA Synthesis, and DNA Extraction

*C. polykrikoides* cultures were harvested by centrifugation at 1,000 g for 10 min, frozen immediately in liquid nitrogen and stored at −80°C until RNA extraction. Preserved cells were physically broken by freeze-thawing in liquid nitrogen and further homogenized by a Mini-Beadbeater (BioSpec Products Inc., Bartlesville, OK) with zirconium beads (diameter 0.1 mm). Total RNA was isolated using the TRIzol (Invitrogen, Carlsbad, CA) and purified by Mini Spin Columns of RNeasy Mini Kit (Qiagen, Valencia, CA). For the first strand cDNA, 2 different cDNA synthesis kits were employed: one was SuperScript III First-Strand Synthesis System (Invitrogen, Carlsbad, CA) for the gene cloning of* CpHSP70* and* CpHSP90*; the other was a* Maxime* RT PreMix Kit with random primers (iNtRON, Seongnam, Republic of Korea) for gene expression study. Then, the 1st strand cDNA templates were diluted 1 : 10 with nuclease-free water for use in subsequent analyses. Total genomic DNA was extracted from* C. polykrikoides* following cetyltrimethylammonium bromide (CTAB) [29].

### 2.3. Gene Sequences Determination

Full length of* CpHSP70* and* CpHSP90* sequences was determined by rapid amplification of cDNA ends (RACE). Partial gene sequences of* CpHSP70* and* CpHSP90* were taken from* C*.* polykrikoides* EST database (GenBank accession number SRR1917383) determined by 454 pyrosequencing (GS-FLX Titanium; 454 Life Sciences, Roche, Branford, CT).* CpHSP70* and* CpHSP90* EST sequences were used for primer design for full length amplification (Table 1). The 3′- and 5′-untranslated regions (UTR) of these genes were determined by using the 3′- and 5′-RACE, respectively. For the RACE, nest PCRs were employed, and the primers used in each PCR were listed in Table 1. Reaction conditions for the primary and secondary PCRs were as follows: predenaturation at 96°C for 10 min; 35 cycles of 95°C for 30 s, 52°C/54°C for 30 s, 72°C for 100 s, and extension at 72°C for 10 min, respectively. Positive core PCR products were purified, cloned into pMD20-T vector (Takara, Shiga, Japan), transformed into* E. coli* competent cells, and subjected to sequencing. The full length of the* CpHSP70* and* CpHSP90* was validated by PCR with specific primers (Table 1). The primers used in the* CpHSP70* and* CpHSP90* genomic sequence determination were designed according to cDNA sequence (Table 1).

### 2.4. *CpHSP70* and* CpHSP90* Characterization and Phylogenetic Analysis

Protein motifs and conserved domains of* CpHSP70* and* CpHSP90* protein were analyzed with the online servers and public database, including the PROSITE (http://prosite.expasy.org/), Compute pI/Mw tool (http://web.expasy.org/compute_pi/), and NCBI Conserved Domain Database (http://www.ncbi.nlm.nih.gov/Structure/cdd/wrpsb.cgi).

Phylogenetic analysis was performed in MEGA5 [30], using the neighbor-joining method [31]. Bootstrap consensus tree inferred from 1,000 replicates was taken to represent the evolutionary history of the taxa analyzed [32]. The tree is drawn to scale, with branch lengths in the same units as those of the evolutionary distances used to infer the phylogenetic tree. The evolutionary distances were computed using the JTT matrix-based method [33] and were in the units of the number of amino acid substitutions per site. In the sequences analysis, all positions containing gaps and missing data were eliminated. It involved 28 amino acid sequences and had a total of 489 positions in the final HSP90s dataset. In the case of HSP70s, it involved 23 amino acid sequences. There were a total of 586 positions in the final HSP70s dataset.

### 2.5. Toxicant Treatments, Gene Expression, and Statistical Analysis

Exponential phase cells were used for toxicant treatments. Typical toxicants CuSO_4_ (Cat. number C1297, Sigma, MO), NaOCl (Cat. number 425044, Sigma, MO), and Aroclor 1016 (48701, Sigma, a type of PCBs) were employed in the present study. To test the doses effect of toxicants on* CpHSP70* and* CpHSP90* transcriptional expression, a series of concentrations of each toxicant were added in the* C. polykrikoides* cultures (with final concentration of CuSO_4_: 1, 5, and 8 mg L^−1^; NaClO_3_: 0.02, 0.1, 0.3, and 0.5 mg L^−1^; PCB: 0.05, 0.1, 0.2, and 0.5 mg L^−1^). The treated and untreated cultures were harvested for gene expression analysis at indicated time points. RNA extraction and cDNA were prepared with the same manner described previously. Gene expression and statistical analysis were followed by Guo et al. [27].

## 3. Results and Discussion

### 3.1. *CpHSP90* Characteristics and Phylogeny

**Figure 1:**
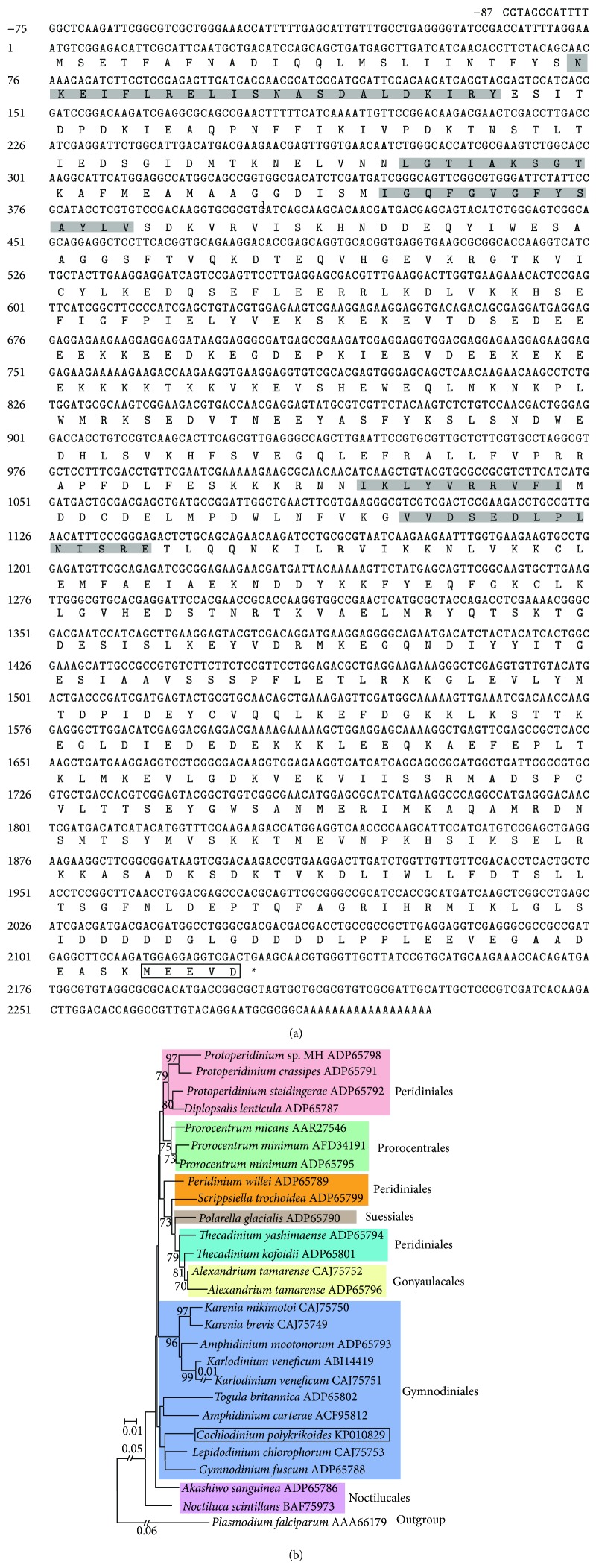
The cDNA and its predicted amino acid sequences of* CpHSP90* and neighbor-joining tree of dinoflagellate HSP90s. (a) The cDNA and its predicted amino acid sequences: the conserved amino acids domains were marked in grey; the end domain “MEEVD” was boxed; (b) a neighbor-joining tree of dinoflagellate HSP90s. The phylogenetic tree was constructed in the MEGA 5 (bootstrap method with 1,000 replicates). The scale bar represents the number of amino acid substitutions per site.* C. polykrikoides *HSP90 was determined in the present study and marked in a box. Other dinoflagellate HSP90s and outgroup were obtained from NCBI database.

*CpHSP90* (GenBank number KP010829) was 2,316 bp in length, coding 709 amino acids (aa) with theoretical isoelectric point (pI) 4.9 and molecular weight (Mw) 81.7 kDa. Its deduced protein shared the highest sequence similarity (681 identities in 709 amino acids) with those of the dinoflagellate* Prorocentrum minimum* (HSP90, GenBank number AFD34191), followed by* Karlodinium veneficum* with 665 identities in 709 aa (HSP90; ABI14419). Generally, the HSP90 contains five conserved motifs defined as HSP90 signature motif [34]; these five signature motifs NKEIFLRELISNASDALDKIRY, LGTIAKSGT, IGQFGVGFYSAYLV, IKLYVRRVFI, and VVDSEDLPLNISRE were identified by comparison with other HSP90s (Figure 1(a)). Furthermore, the conserved MEEVD was identified in the C-terminus of deduced CpHSP90, which indicated that CpHSP90 protein belongs to the cytosolic HSP90 family [35].

Phylogenetic analysis was performed using CpHSP90 protein and other dinoflagellate HSP90 proteins (Figure 1(b)). A resultant tree showed* C. polykrikoides* with* Gymnodinium fuscum* and* Lepidodinium chlorophorum* was clustered into one clade, of which taxon position belonged to the order Gymnodiniales. Furthermore, other dinoflagellate orders like Perdiniales were spited into two clades as well. These results suggested that the dinoflagellate HSP90s explosive scattered in morphology and diversity [24].

### 3.2. *CpHSP70* Characteristics and Phylogeny

**Figure 2:**
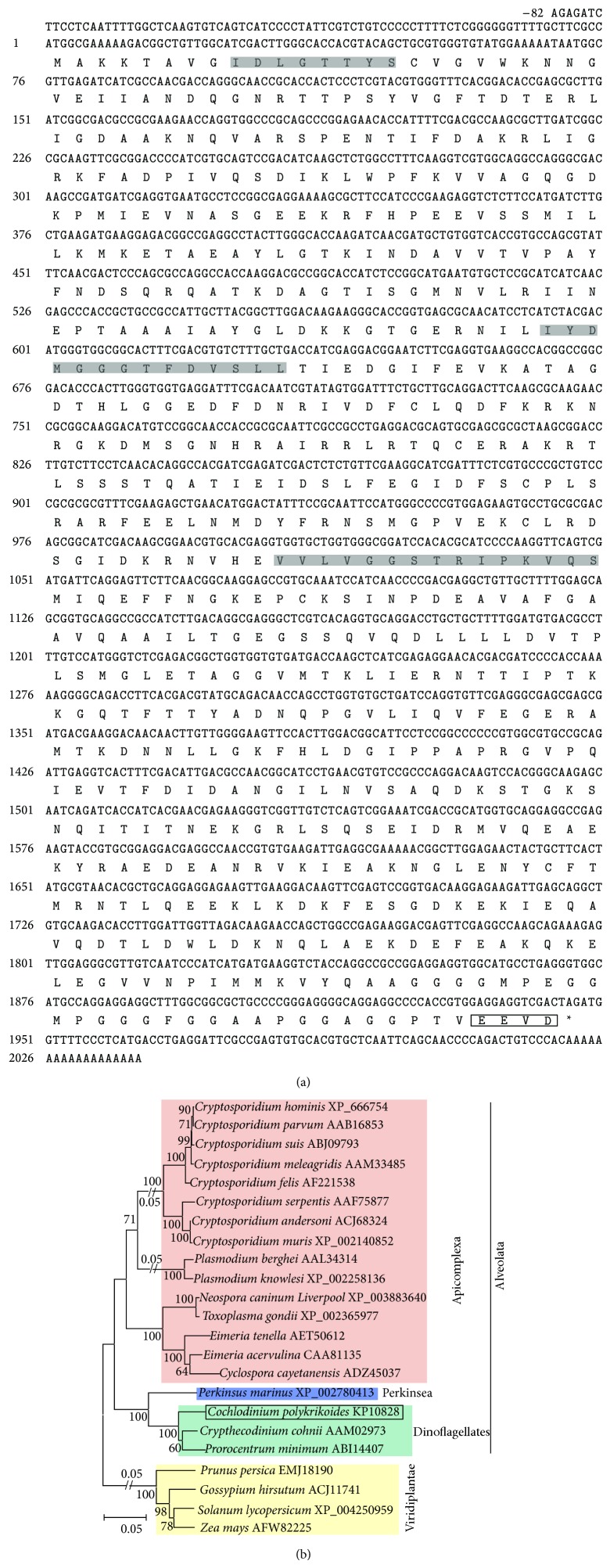
The cDNA and its predicted amino acid sequences of* CpHSP70* and neighbor-joining tree of dinoflagellate HSP70s. (a) The cDNA and its predicted amino acid sequences: the conserved amino acids domains were marked in grey; the end domain “EEVD” was boxed; (b) the phylogenetic tree was constructed in the MEGA 5 (bootstrap method with 1,000 replicates), and the scale bar represents the number of amino acid substitutions per site.* CpHSP70* was determined in the present study and marked in a box. Other dinoflagellates HSP70s and outgroup were obtained from NCBI database.

In addition, full ORF of* CpHSP70* (GenBank number KP010828) was 1,944 bp in length, coding 648 aa with theoretical pI 5.12 and Mw 70.8 kDa.* CpHSP70* aa showed 94% maximum identity with those of the dinoflagellates* Crypthecodinium cohnii* (GenBank number AAM02973) and* Prorocentrum minimum* (ABI14407), followed by 88% identity with* Perkinsus marinus* (XP_002780413). We identified three HSP70 motifs, IDLGTTYS, IYDMGGGTFDVSLL, and VVLVGGSTRIPKVQS, in this protein (Figure 2(a)). In addition, the EEVD motif was identified in the CpHSP70 protein C-terminus, which indicated that the CpHSP70 located in the cytoplasm of the cell [36].

A neighbor-joining tree was constructed using dinoflagellates HSP70 and other eukaryotic HSP70s (Figure 2(b)). As expected, all the dinoflagellate HSP70s were clustered into one clade, which showed closest relationship with Perkinsea, followed by Apicomplexa. All the analyzed dinoflagellates, Perkinsea, and Apicomplexa were grouped into one clade belonging to Alveolata.

### 3.3. The Genomic Coding Structures of* CpHSP70* and* CpHSP90*

**Figure 3:**
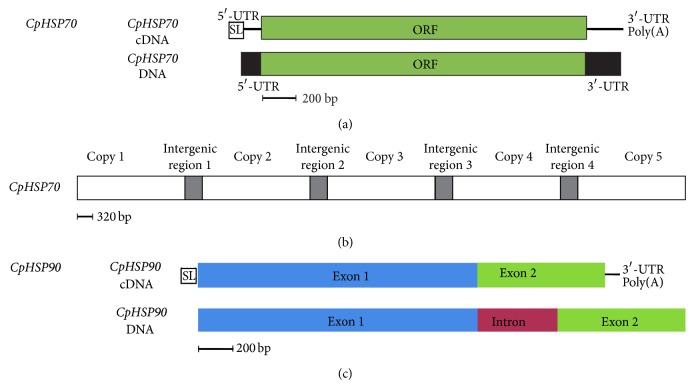
Schematic structures of genomic DNA of* CpHSP70* and* CpHSP90*. (a) Comparison of DNA and cDNA of* CpHSP70*. (b) Predicted genomic DNA structure of the* CpHSP70*: the different copies of the* CpHSP70* genes are separated by intergenic regions. (c) Comparison of DNA and cDNA of* CpHSP90*. One intron (red box) was found in the genomic DNA sequence of* CpHSP90*.

Genomic regions of each* CpHSP70* and* CpHSP90* were amplified by PCR. As a result, we found that no intron was presented in the* CpHSP70* (KP010830) coding genome. In addition, the gene was encoded in tandem arrangement manner with the separation of intergenic spacers (Figures 3(a) and 3(b)), which was 397 bp in length, and was found in* CpHSP70* genome sequence. This result was similar to that of* Amphidinium carterae* [37]. On the other hand, interestingly, we found one intron as in* CpHSP90* coding genome (KP010831) (Figure 3(c)), which was 454 bp in length, but no intergenic region was found, as judged by PCR. This structure was different from that of* A. carterae* HSP90 genomic sequence (25 introns), and* A. carterae HSP90* gene was encoded in tandem arrangement. Although* A. carterae HSP90* and* CpHSP90* proteins showed highly homologous phylogenic relationships, their genomic DNA represented quite different characteristics. These results suggested that the same gene in different dinoflagellates displayed differential genome arrangement.

### 3.4. Effect of Algicide on* CpHSP70* and* CpHSP90* Transcription

Algicide chemical treatments are one of the powerful tools to remove HABs, and the physiological effects of individual algicides on the HABs have been widely investigated [38]. For example, the algicides oxidizing NaOCl and nonoxidizing CuSO_4_ can cause considerable decreases of* C. polykrikoides* cell numbers and pigment contents and also affect their chlorophyll autofluorescence [38]. Nevertheless, there is no report about the CuSO_4_ and NaOCl effect on the* C. polykrikoides* at molecular level, especially in terms of gene transcription.

**Figure 4:**
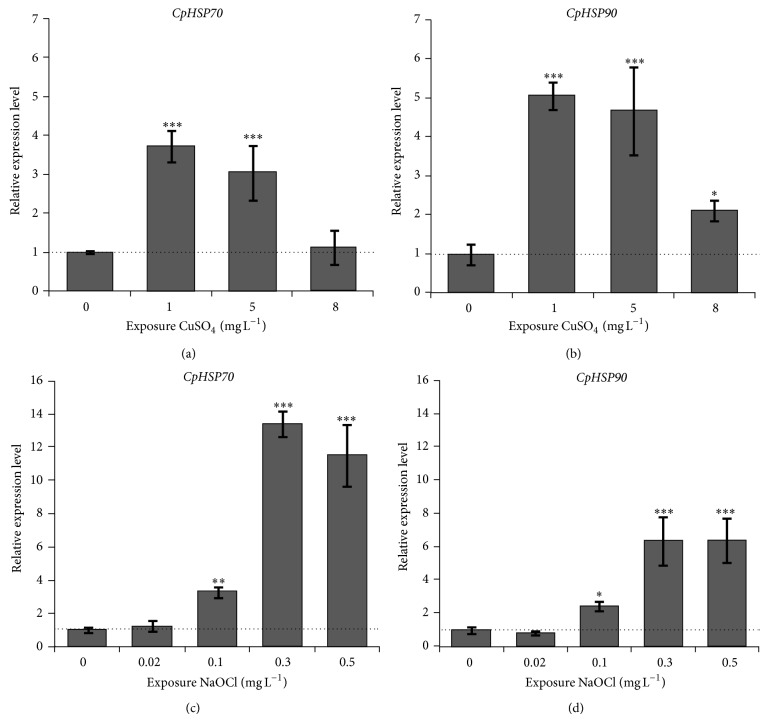
The gene expression profile of* CpHSP70* and* CpHSP90* after CuSO_4_ and NaOCl treatment, respectively. (a, b) Total RNA was extracted from* C. polykrikoides* at 24 h after treatment with 1 mg L^−1^, 5 mg L^−1^, and 8 mg L^−1^ of CuSO_4_, respectively. (c, d) Total RNA was extracted from* C. polykrikoides* after treatment with 0.02 mg L^−1^, 0.10 mg L^−1^, 0.3 mg/L, and 0.5 mg L^−1^ NaOCl, respectively. The* 18S rRNA* gene was used as the internal control to normalize the amount of templates in qRT-PCR. Results are given as the means of triplicate ±SD. The significant differences between the treated group and the control group are highlighted by one-way ANOVA; ^∗^ *P* < 0.05, ^∗∗^ *P* < 0.01, ^∗∗∗^ *P* < 0.001.

In general, effects of the CuSO_4_ on aquatic organisms are relative to the formation of reactive oxygen species (ROS) and can regulate the photosynthesis related gene expression and increase the antioxidant enzyme activity in the algae [39, 40]. In the present study, the transcriptional expression of* CpHSP90* and* CpHSP70* showed similar expression pattern after CuSO_4_ treatment (Figure 4). The transcriptional expression level of these two genes was first upregulated and then decreased with increased concentration of CuSO_4_. The* CpHSP90* transcription showed similar expression pattern under 1.0 mg L^−1^ and 5.0 mg L^−1^ CuSO_4_ treatments with 5.1- and 4.7-fold changes compared to that of control, respectively. These expression patterns were also displayed by* CpHSP70*, showing 3.7- and 3.1-fold changes under 1.0 mg L^−1^ and 5.0 mg L^−1^ CuSO_4_ treatments compared to that of control. Either* CpHSP90* or* CpHSP70* was distinctly decreased compared to those of 1.0 mg L^−1^ or 5.0 mg L^−1^ CuSO_4_ treatment (Figures 4(a) and 4(b)). This result suggested that the* CpHSP90* and* CpHSP70* may be involved in CuSO_4_ induced gene regulation in* C. polykrikoides*. This result was congruent with our previous results, showing that both genes were considerably induced by exposure of CuSO_4_ in the dinoflagellate* P. minimum* [27, 28]. However, we found that the expression patterns of* HSP70* genes were different between* C. polykrikoides* and* P. minimum* (Pm) exposed to the same CuSO_4_. The* CpHSP70* expression was increased gradually till high dose (8.0 mg L^−1^ of CuSO_4_), whereas the* PmHSP70* expression was the highest level at 1.0 mg L^−1^ and then decreased, possibly due to cell deaths. According to these results, we predicted that the* HSP90* and* HSP70* are commonly involved in metal CuSO_4_ induced gene regulation in the dinoflagellates, but their expression patterns may depend on exposed doses and testing species.

Oxidizing chlorine is one of biocides that are commonly used in controlling the quality of the water. It can damage the cell by producing ROS. Hypochlorites may have primary deleterious effect on the DNA synthesis or progress oxidation of thiol groups and further effect on the cell wall and protein [41]. In the present study, the expression pattern showed response of* CpHSP90* and* CpHSP70* depending on the doses of the NaOCl. Interestingly,* CpHSP90* and* CpHSP70* were not induced by lower concentration (0.02 mg L^−1^) of NaOCl but significantly upregulated by relatively high concentrations (0.1, 0.3, and 0.5 mg L^−1^) of NaOCl (Figures 4(c) and 4(d)). Both* CpHSP90* and* CpHSP70* in 0.3 mg L^−1^ NaOCl treated cells showed 8.5- and 13.4-fold changes compared to that of control, which were highest expression level among NaOCl treated cells. The chlorine-based disinfectants induced HSP70 gene expression was also found in the* C. parvum* oocysts [42].

### 3.5. Effect of PCB on* CpHSP70* and* CpHSP90* Transcription

There are many of chemicals that presented in the aquatic system by industry or agriculture sewerage. PCBs are used in the industry and commonly present in aquatic ecosystems. They are one of the endocrine disturbing chemicals (EDCs) and have toxic effect to various organisms [43]. According to our previous work [44], EDCs, including PCB, were very toxic to microalgae, potentially affecting the photosystem II energy flow, of which results suggested their toxic effect on the dinoflagellates.

**Figure 5:**
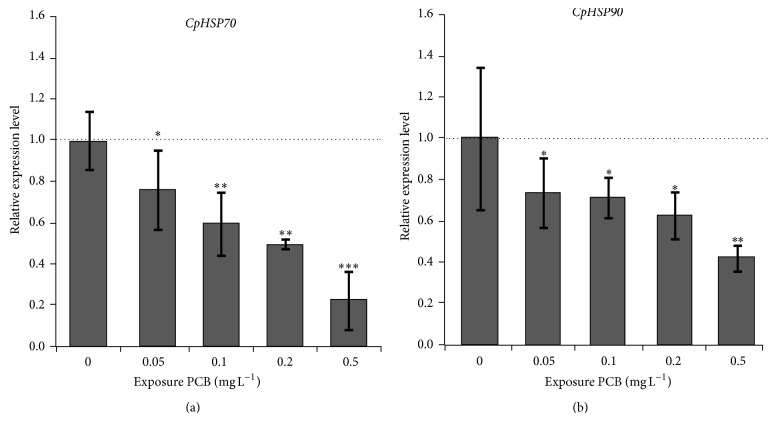
Gene expression profile of* CpHSP70* (a) and* CpHSP90* (b) after PCB treatment, respectively. Total RNA was extracted from* C. polykrikoides* at 24 h after treatment with 0.05 mg L^−1^, 0.1 mg L^−1^, 0.2 mg L^−1^, and 0.5 mg L^−1^ of PCB, respectively. The* 18S rRNA* gene was used as the internal control to normalize the amount of templates in qRT-PCR. Results are given as the means of triplicate ±SD. The significant differences between the treated group and the control group are highlighted by one-way ANOVA; ^∗∗^ *P* < 0.01, ^∗∗∗^ *P* < 0.001.

In this study, we examined the gene expressional response of* CpHSP70* and* CpHSP90* in* C. polykrikoides* exposed to PCB. The transcriptional expressions of* CpHSP70* and* CpHSP90* were gradually decreased with increasing PCB concentration (Figure 5), with lowest expression level of 0.42- and 0.23-fold under 0.5 mg L^−1^ PCB exposure compared to untreated cultures. However, neither* PmHSP90* nor* PmHSP70* was up- or downregulated by the PCB exposure in the dinoflagellate* Prorocentrum minimums* [27, 28]. According to these results, we speculated that the PCB may have a differential effect on the dinoflagellates among species. The aroclor 1016, which is one of dioxin-like PCBs, was employed. It is clear that the aryl hydrocarbon receptor (Ahr) mediated pathway is involved in the dioxin-like PCBs adverse effect mechanism [45, 46]. A dimer of HSP90 is essential compound that binds to the inactive Ahr protein in the cytoplasm and needs to be released from Ahr protein complex when Ahr is activated [47–50]. According to these findings, we predict that the* CpHSP90* may bind to Ahr and express at a high level in the normal conditions. The* CpHSP90* may be released, and then the protein may be activated to participate in the signal transduction of the cells, when* C. polykrikoides* is exposed to PCB. The essential* CpHSP90* amount was decreased with increasing concentration of PCB in the* C. polykrikoides*. At present, it was not clear; thus we needed more experiments to clarify these gene responses in the dinoflagellate, by using additional EDCs and testing species in future.

In addition to this, the* CpHSP70* and* CpHSP90* showed similar expression patterns under the same PCB treatment (Figures 4 and 5). Similar results were found in the benzo[*a*]pyrene treated clam* Ruditapes philippinarum* [51]. Both* CpHSP90* and* CpHSP70* proteins contain EEVD motif, which is tetratricopeptide repeat domain binding site at the C-terminus, and some proteins can bind with HSP90 and HSP70 to assemble as protein complex to play function such as in the triage of damaged and aberrant proteins for degradation process [16, 52]. The gene transcription pattern of* CpHSP90* and* CpHSP70* results implied these two genes may have cooperative function in the* C. polykrikoides* toxicant induced gene regulation [16, 52].

In conclusion, this study firstly determined full length cDNAs of two HSPs (HSP70/90) from the harmful dinoflagellate* C. polykrikoides* and characterized molecular features such as conserved motifs, coding genomic region, and phylogenetic relatedness to other eukaryotes.* CpHSP70* had quite similar cDNA and genomic coding structures (e.g., no intron and tandem arrangement) to those in the other dinoflagellates; however,* CpHSP90* was different from those of other dinoflagellate HSP90s in coding genomic structure (one intron and no intergenic region). These suggested their homologous functions with difference of genomic DNA evolutionary events. In addition, both* CpHSP90* and* CpHSP70* may be involved in responding to the CuSO_4_, NaOCl, and PCB caused stress.

## Figures and Tables

**Table 1:** The primers used in the present study.

Gene	Primer	Remark	Nucleotide sequence (5′ → 3′)
*CpHSP90 *	CpH90F1	RT-PCR	GTTCGACACCTCACTGCTCACC
*CpHSP90 *	CpH90R1	RT-PCR	AGGCCGAGCTTGATCATGC
*CpHSP70 *	CpH70F1	RT-PCR	GGGCAGACCTTCACGACGTATG
*CpHSP70 *	CpH70R1	RT-PCR	AATGCCGTCCAAGTGGAACTTC
*Cp18S *	Cp18S-F	RT-PCR	GGAGTATGGTCGCAAGGCTGAAAC
*Cp18S *	Cp18S-R	RT-PCR	CCTCGTGTTGAGTCAAATTAAGCC
*CpHSP90 *	CpHSP90-3F2	3′-RACE	TGCTCACCTCCGGCTTCAAC
*CpHSP90 *	CpHSP90-3F3	3′-RACE	CCACCGCATGATCAAGCTC
*CpHSP90 *	CpHSP90-5R2	5′-RACE	ATGCGGCTGCTGATGATG
*CpHSP90 *	CpHSP90-5R3	5′-RACE	ACCTTCTCCACCTTGTCGC
*CpHSP90 *	CpHSP90-SR1	Full length	GTGTCCAAGTCTTGTGATCGAC
*CpHSP90 *	CpHSP90-SR2	Full length	AGCACTAGCGCCGGTCATGT
*CpHSP90 *	5-SL	5′-RACE/full length	CGTAGCCATTTTGGCTCAAG
*CpHSP70 *	CpHSP70-3F1	3′-RACE	CGCTGCAGGAGGAGAAGTTG
*CpHSP70 *	CpHSP70-3F2	3′-RACE	TGGAGGGCGTTGTCAATC
*CpHSP70 *	CpHSP70-SF1	Full length	TTTCTCGGGGGTTTTGCTTCG
*CpHSP70 *	CpHSP70-SF2	Full length	CATGGCGAAAAAGACGGCTGTT
*CpHSP70 *	CpHSP70-SR1	Full length	TGGGACAGTCTGGGGTTGCT
*CpHSP70 *	CpHSP70-SR2	Full length	ACACTCGGCGAATCCTCAG
	B26	3′-RACE	GACTCTAGACGACATCGA(T)_18_
	B25	3′-RACE	GACTCTAGACGACATCGA
*CpHSP70 *	CpHSP70-DF1	Genomic DNA	TGTCAGTCATCCCCTATTTGTC
*CpHSP70 *	CpHSP70-DR1	Genomic DNA	GACAGTCTGGGGTTGCTGAATT
*CpHSP90 *	CpHSP90-DF1	Genomic DNA	TGGGAAACCATTTTTGAGCATTG
*CpHSP90 *	CpHSP90-DR1	Genomic DNA	TGCAGGTGTGAACCACTCAGC
*CpHSP70 *	CpHSP70-IF1	Intergenic DNA	GAGGGCGTTGTCAATCCCATG
*CpHSP70 *	CpHSP70-IR1	Intergenic DNA	AATGCCGTCCAAGTGGAACTTC
